# Biological age and tempos of aging in women over 60 in connection with their morphofunctional characteristics

**DOI:** 10.1186/1880-6805-33-12

**Published:** 2014-05-19

**Authors:** Marina Negasheva, Natalia Lapshina, Rostislav Okushko, Elena Godina

**Affiliations:** 1 Department of Anthropology, Faculty of Biology, Lomonosov Moscow State University, Leninskie Gory, 1, Bld. 12, Moscow 119234, Russia; 2 School of Medicine, Shevchenko Transdniestrian State University, 25th October str., 93, Tiraspol MD-3300, Pridnestrovian Moldavian Republic; 3 Institute and Museum of Anthropology, Moscow State University, Mokhovaya Str., 11, Moscow 125009, Russia

**Keywords:** Biological age, Tempos of aging, Body build, Body composition, Elderly and long-lived women

## Abstract

**Background:**

The study of aging processes and the changes in morphological, physiological, and functional characteristics that are associated with aging is of great interest not only for researchers, but also for the general public. The aim of the present paper is to study the biological age and tempos of aging in women older than 60 years, including long-lived females (over 90-years-old), and their associations with morphofunctional characteristics.

**Results:**

Somatic traits, body mass components, and functional characteristics were investigated in 119 elderly (between 60 and 74-years-old) and long-lived (over 90-years-old) women in Tiraspol. With the special PC software ‘Diagnostics of Aging: BioAge’ (National Gerontological Center, Moscow, Russia) the biological age and tempos of aging were evaluated in the study participants. The results show close connections between morphofunctional changes, particularly in body mass components, and biological age. The software demonstrated its validity in the estimation of biological age in the group of elderly women. In the homogenous (according to their chronological age) group of women, three subgroups were separated with different tempos of aging: those with lower rates of aging (biological age less than chronological age by two years or more); those consistent with their chronological age, and those with accelerated tempos of aging (biological age higher than chronological age by two years or more).

**Conclusions:**

Morphofunctional characteristics in the studied groups of women demonstrate the trends of age-involutive changes which can be traced through all groups, from those with slow rates of aging, to those with average rates, to those with accelerated tempos of aging, and finally in long-lived women. The results of comparative analysis show that women with accelerated aging are characterized with such traits as lower skeletal muscle mass, lower hand grip strength, and higher metabolic rate. Canonical discriminant analysis revealed a number of morphofunctional characteristics which differentiate the early-aging women from women with average rates of aging: higher BMI values, excessive fat mass, lower skeletal muscle mass and low values of hand grip strength. Thus the presence of such characteristics in elderly women can be considered as additional risk factor towards the early onset of the aging process.

## Background

The study of aging processes and of the changes in morphological, physiological and functional characteristics that are associated with aging is of great interest not only for researchers in anthropology, gerontology, and medicine [1-12], but also for the majority of people that have little to do with science but have stepped over a certain age boundary and moved into senior age groups (for example 55 to 60-year–olds).

The steady growth of the proportion of elderly people in the population of some countries and low life-expectancy in other countries (including Russia) necessitate searching for new high-tech approaches aimed at increasing people’s lifespans, both physically and mentally. It is also important to find adequate techniques for evaluating the health status, profile, and tempos of aging in the modern population.

Senescence is characterized by an aging decrease in the structural order of the organism, by an increasing degree of its wear, decreasing vitality, functional capacities, and adaptability, as well as an increasing probability of disease and death from a number of causes [2,3,9]. At the same time, the rate of involutional processes differs not only among the various systems of the organism, thus revealing the effects of the heterochrony of aging, but also among individuals. To study the individual variations in the tempos of aging the notion of biological age has been introduced, which is determined by factors such as physiological changes rather than chronology.

Biological age (BA) is an indicator of development, changes or wear, and loss of the structures and functions of some systems or the organism as a whole. BA is a fundamental characteristic of the tempos of aging. It is determined by a set of metabolic, structural, functional, regulatory, and adaptive properties of the organism. As a model concept, it consists of a certain compliancy between the individual level of morphofunctional status and a certain average for a given population [9,13-16].

Today there are roughly two dozen methods for the evaluation of biological age, using different indicators regularly changing with age [14,16-21]. Among the most recent convenient, simple, low cost, and quick techniques are the H-Scan testing method [14,22,23] and the package ‘Diagnostics of Aging: BioAge’ (National Gerontological Center, Moscow, Russia, http://www.ngcrussia.org/) [7,16,17].

In works by Anisimov [1] and Schulz-Aellen [11], among many others, a huge spectrum of modern theories on the molecular and physiological mechanisms of aging and senescence are given. Many authors explain the acceleration of senescence by the development of some diseases and pathological processes [4,24], while others think more about social factors and climatic conditions [25,26].

The present paper is aimed at the study of biological age and the tempos of aging in women older than 60 years, including long-lived females, and their associations with morphofunctional characteristics.

## Materials and methods

This study is based on the integrated medical-anthropological investigation of 70 women aged 60 to 74-years-old, and 49 long-lived women (from 90 to 104-years-old), all inhabitants of Tiraspol (Pridnestrovian Moldavian Republic). The study has taken place in a holiday centre of general (not medical) orientation. Women with serious chronic illnesses, such as ischemic heart disease or diabetes, were not included in the studied sample. The survey was performed in the autumn of 2012 by the staff of the Biological Faculty, Lomonosov Moscow State University, in collaboration with the Medical Faculty of the Shevchenko Transdniestrian State University. The subjects were mainly Russians (about 15% were Moldovians).

The program included several procedures and collected many different variables of data. Anthropometric measurements (height and weight, waist and hip circumferences) were taken. Standing height was measured using a Model 101 – Anthropometer (GPM manufacturers, Switzerland, http://www.seritex.com/gpm) and weight was measured on a digital scale. Both circumferences were measured using a measuring tape. Body mass index (BMI) was calculated as body mass (BM) divided by standing height (Ht) squared. The whole-body impedance was measured on the right hand side of the body using the bioimpedance meter ABC-01 ‘Medas’ (SRC Medas, Russia) according to a conventional tetrapolar scheme at a frequency of 50 kHz. Phase angle (PA) was calculated as:

$$\arctan\;\left(\mathrm{XC}/R\right)*180{}^{\circ}/p$$

where *XC* is the reactance and *R* the whole-body electric resistance. Fat-free mass (FFM) was assessed using the Houtkooper equation [27]:

$$\mathrm{FFM}=0.61*\left(\mathrm{Ht}2/R\right)+0.25*\mathrm{BM}+1.31$$

where Ht is measured in cm. Fat mass (FM) was calculated as the difference between BM and FFM, and %FM as:

$$\left(\mathrm{FM}/\mathrm{BM}\right)*100$$

Other body composition variables, such as body cell mass (BCM) and skeletal muscle mass (SMM), were determined by analogy using appropriate regression formulas provided by the manufacturer. With the bioimpedance meter it was also possible to measure one of the most important characteristic of human metabolism, the basal metabolic rate (BMR), defined as the minimal amount of energy necessary for the maintenance of vital bodily functions when at rest. To compare the intensity of metabolic processes in different individuals, the values of BMR are given per 1 m^2^ of body surface (specific metabolic rate) [28,29]. Functional characteristics of cardiovascular systems were measured: systolic and diastolic blood pressure (mmHg) and heart rate (beats per minute). Hand grip strength for right and left hands was measured with the hand dynamometer - DK 50. Estimation of BA was performed with the software ‘Diagnostics of Aging. BioAge’ (National Gerontological Center, Moscow, Russia, http://www.ngcrussia.org/), and included the set of functional biomarkers of the cardiovascular and respiratory systems [7,16]: systolic and diastolic blood pressure, forced lung capacity (ml), expiratory breath-holding (seconds), crystalline accommodation (the distance of the nearest viewpoint in diopters), acuity of hearing or auditory threshold under 4,000 Hz (dB), body mass (kg), self-evaluation of health status (the number of negative answers to 29 standard questions), Wechsler Adult Intelligence Test (the number of correctly filled cells per 90 seconds) and others.

A direct comparison of the estimated BA and real chronological age is not consistent. For young people BA could be overestimated, and for senior people it could be underestimated. To compensate for this inconsistency, estimated BA values should be compared with the values of so-called ‘proper biological age’ (PBA) [7,16,18,23], which is calculated as a linear function of the chronological age according to the following formula:

$$\mathrm{PBA}=\left(CA_{\mathrm{ind}}-CA_{\mathrm{av}}\right)*R^{2}+CA_{\mathrm{av}},$$

Where PBA is the proper biological age, CA_ind_ is the individual chronological age, CA_av_ is the average chronological age of the subjects, and R is the multiple correlation coefficient of tests results with the chronological age.

In this study the CA_av_ for 60 to 74-year-old women was equal to 66.4 years and the multiple correlation coefficient was equal to 0.988.

The BA estimated for each individual was compared with the PBA value: If BA = PBA ± 2 years – an individual is characterized by the average for this population tempos of aging;

If BA < PBA – 2 years - an individual is characterized by slow tempos of aging;

If BA > PBA + 2 years - an individual is characterized by accelerated tempos of aging.

Statistical analysis was performed with the Statistica (v. 6.0) software (Statsoft Inc., Tulsa, USA). Student’s *t*-test was used for comparison of means in two subsamples. To compare the means of morphological and functional parameters in several groups of women (with different tempos of aging) one-way analysis of variance (ANOVA) was used. Intergroup variability of the set of morphofunctional characteristics in women with different tempos of aging was studied with canonical discriminant analysis.

Research was carried out in compliance with the Helsinki Declaration, approved by Bioethical Committee of Medical School, Transdestrian State University, protocol # 215.03/12.

## Results and discussion

In Table 1 means and standard deviations for anthropometric measurements and body mass components are presented for two groups of study participants.

**Table 1:** Body parameters of women of different age

**Traits**	**Elderly women (60 – 74 years), N = 70**		**Long-lived women (90 – 104 years), N = 49**	
	**Mean**	**SD**	**Mean**	**SD**
Body mass (kg)	79,9	14.8	58.7	11.7
Height (cm)*	158.1	6.1	148.7	7.3
BMI, kg/m^2^*	31.9	5.2	26.5	4.5
Waist circumference (cm)	97.7	12.3	90.1	11.8
Hip circumference (cm)	111.3	10.3	97.7	9.6
Ratio: waist circumference/hip circumference*	0.88	0.06	0.92	0.09
Fat mass (kg)*	32.2	10.7	16.7	7.7
Skeletal muscle mass (kg)*	19.2	2.3	16.1	5.2
Active cell mass (kg)	25.8	3.4	24.0	7.5

*statistically significant (*P* <0.05). BMI, Body Mass Index; SD, Standard deviation.

In the study elderly women between 60 and 74-years-old have very high BMI values, which correspond to overweight and even first degree obesity. These results coincide with literary data concerning high BMI values in Russian women of this age group [30,31]. The mean values of body mass, and particularly of BMI, are much higher in the group of elderly women, when compared with the long-lived group (see Table 1).

For women over 90 the BMI values are significantly lower, which corresponds with data published by other authors [32-34]. There are statistically significant differences in height for the group of elderly women and the long-lived group who is characterized with the lowest values of this trait (*P* <0.05).

The results of the bioimpedance analysis show the differences in the values of body mass components in women of the two studied age groups (see Table 1). The amount of fat mass in elderly women is twice as much as that in the longevity group. At the same time, in women over 90 the amount of skeletal muscle mass is statistically lower, while the decrease of body cell mass is not significant: 25.8 kg in the group of elderly women versus 24.0 kg in the longevity group.

Based on these results it is possible to assume that the aging process includes the decrease of body mass primarily at the expense of fat mass, and to a lesser extent at the expense of the muscle component. As the values of the active cell mass are practically the same in both groups studied, it may serve as evidence of a high level of physical activity in women of the longevity group. The active cell mass is an indirect indicator of the active way of life as it reflects the content of metabolically active tissues [28].

There are many studies showing the changes in BMI, fat, and muscle body mass components connected with age and the way of life [35-38]. Their results are comparable with those in the present study. However, such studies for the long-lived group are extremely rare and rather contradictory [39].

The ratio of waist and hip circumferences is a somatic characteristic which reflects sexual dimorphism in body build and shape. It can tentatively be called the index of andromorphy and gynomorphy [9]. For women the most typical values of this index lie in the range between 0.6 and 0.8, which is reflected in a narrow waist and wide hips (pear-shaped physique). Values over 0.8 indicate increasing fat accumulation on the trunk of the body (predominately in the central, abdominal area) and are associated with the changes towards masculine, andromorphic body build, when the subcutaneous fat topography changes from a female type (fat prevailing on hips) to a male one (fat prevailing on the trunk, apple-shaped physique).

The values of this index in the studied women (see Table 1) reflect the accumulation of the fat in abdominal area, which coincides with the data published by other researchers [40,41]. However, in the long-lived group the amount of fat mass is much lower compared to the elderly group, and it can be suggested that women over 90 have a tendency towards a masculine body build (andromorphy).

In Table 2 means and standard deviations of functional parameters are presented for two groups of the studied women.

**Table 2:** Functional characteristics of the cardiovascular system and hand grip strength values in women of different age groups

**Traits**	**Elderly women (60 – 74 years), N = 70**		**Long-lived women (90 – 104 years), N = 49**	
	**Mean**	**SD**	**Mean**	**SD**
Systolic blood pressure* (mmHg)	149.6	19.7	159.3	28.3
Diastolic blood pressure (mmHg)	80.6	11.8	86.2	21.5
Pulse rate* (beats per minute)	72.1	11.0	82.4	14.3
Right hand strength* (kg)	21.6	6.2	9.4	4.0
Left hand strength* (kg)	19.5	6.1	8.3	3.8
Specific metabolism*	809.0	61.8	938.8	73.6

*statistically significant (*P* <0.05).

The analysis of the functional characteristics of cardiovascular system and hand grip strength in the two groups of women (see Table 2) showed statistically significant differences. In women of the long-lived group, mean values of systolic blood pressure and heart rate were increased. This was also observed by other authors [42].

In women over 90 there were also noticeable differences in their strength characteristics. The grip strength for both hands was almost twice as less as in the elderly women. This is particularly interesting because the difference in skeletal muscle mass was not that significant (see Table 1) [43,44].

After the BA values were received for all women, they were divided into three groups according to the tempos of aging: slow aging, average aging and accelerated aging. The average chronological age in each of the three groups was approximately 66 years. The fourth subgroup was made up of the long-lived subjects. Means and standard deviations of anthropometric measurements and body mass components for the studied women are presented in Table 3.

**Table 3:** Somatic traits in women with different tempos of aging

**Traits**	**Slow aging, N = 32**		**Average aging, N = 23**		**Accelerated aging, N = 15**		**Long-lived women, N = 49**	
	**Mean**	**SD**	**Mean**	**SD**	**Mean**	**SD**	**Mean**	**SD**
Body mass (kg)	83.5	16.1	77.8	13.4	75.3	12.7	58.7	11.7
Height (cm)	159.3	6.2	158.3	6.7	155.1	4.0	148.8*	7.3
BMI, kg/m^2^	32.9	5.6	31.1	4.8	31.3	4.9	26.5*	4.5
Waist circumference (cm)	98.6	13.5	96.0	12.1	98.9	10.6	90.1	11.8
Hip circumference (cm)	112.6	12.0	109.8	8.5	111.0	9.1	98.0	9.6
Waist-hip ratio	0.88	0.07	0.87	0.05	0.89	0.06	0.92	0.09
Fat mass (kg)	34.7	11.8	30.3	8.9	29.6	10.4	16.7*	7.7
Skeletal muscle mass (kg)	19.7	2.6	19.3	2.7	18.1	2.2	16.1	5.2
Active cell mass (kg)	26.5	3.3	25.3	3.5	25.1	3.5	24.0	7.5

*statistically significant (*P* <0.05) when the means of body parameters for group 4 (long-lived women) are pairwise compared with all other groups. BMI, Body Mass Index; SD, Standard deviation.

When groups of women with different tempos of aging were compared, a steady gradient of changes in the somatic development of women was revealed, from the group of slow aging women to the long-lived women (see Table 3). It is particularly interesting to compare those changes for the three groups of women with different biological ages (accordingly with different tempos of aging), as the chronological age was the same (66 years) in those groups and the indices of body mass components were not taken into account for the estimation of biological age.

For women with the accelerated tempos of aging, a clear decrease of the amount of skeletal muscle mass and active cell mass was shown, which was particularly significant for the fat mass. This may serve as an evidence of the acceleration of the involutive age changes of somatic development in this group.

In Table 4 means and standard deviations of functional parameters are presented for the groups of the studied women.

**Table 4:** Functional characteristics in women with different tempos of aging

**Traits**	**Slow aging, N = 32**		**Average aging, N = 23**		**Accelerated aging, N = 15**		**Long-lived women, N = 49**	
	**Mean**	**SD**	**Mean**	**SD**	**Mean**	**SD**	**Mean**	**SD**
Systolic blood pressure (mmHg)	147.3	21.0	151.9	18.9	151.0	18.8	159.3	28.3
Diastolic blood pressure (mmHg)	77.3	10.9	83.6	13.5	82.6	9.1	86.1	21.5
Pulse rate (beats per minute)	69.7	8.7	75.2	13.3	72.1	10.8	82.4*	14.3
Right hand strength (kg)	24.9*	4.8	19.9	5.5	16.8	6.1	9.4*	4.0
Left hand strength (kg)	22.6*	5.8	18.5	4.6	14.4	6.0	8.3*	3.8
Specific metabolism	799.2	57.9	806.5	44.0	835.6	88.6	938.8*	73.6

*statistically significant (*P* <0.05) when the means in the assigned groups are compared with the other groups.

The functional characteristics of the cardiovascular system and the hand grip strength were included in the evaluation of the biological age, and their decrease from the slow aging group to the long-lived group is evident (see Table 4). However, it is interesting that the specific metabolic rate increases from the slow aging group to the long-lived group. This may be considered as evidence of the intensification of compensatory adaptive mechanisms in the process of aging.

Thus, in women with accelerated aging there are typical age changes in different systems of the organism (body mass components, functional traits) that show earlier signs of involutive processes, indicating a tendency to an earlier transition to senescence for these women in spite of their chronological age. Although the average chronological age in this group of women is 66 years, their morphofunctional characteristics are very close to those of women from the long-lived group. Women from the accelerated group are in need of a serious medical examination to find out the causes of their early aging, and the means to correct it.

At the final stage, multivariate canonical analysis was used with the following variables: heart rate, hand grip strength, waist-hip ratio, fat and skeletal muscle mass, and specific metabolic rate (Table 5).

**Table 5:** Standardized coefficients of morphofunctional characteristics as canonical variables

**Traits**	**Canonical variables**	
	**К**_**1**_	**К**_**2**_
Pulse rate	0.25	0.49
Right hand strength	-0.77	0.32
Left hand strength	-0.72	0.57
BMI	-0.32	-0.38
Waist-hip ratio	0.16	0.02
Fat mass	-0.48	-0.42
Skeletal muscle mass	-0.25	0.07
Specific metabolic rate	0.33	0.19

**Figure 1:**
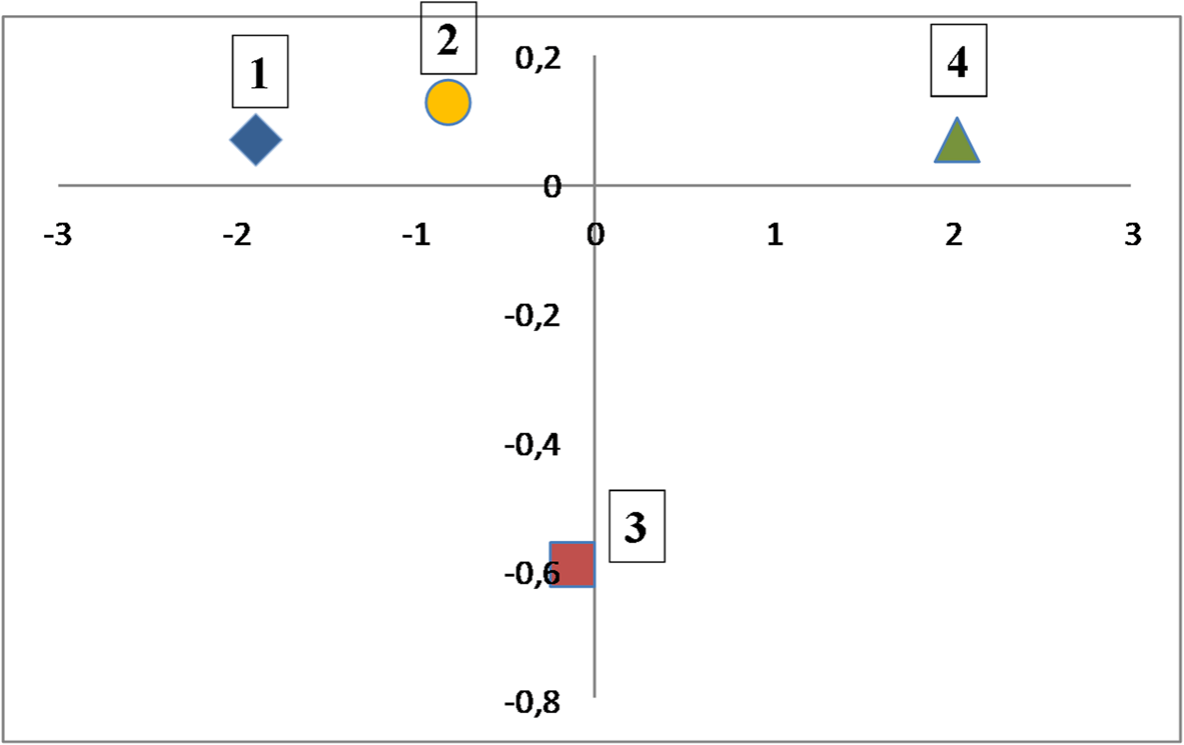
The results of canonical analysis for women with different tempos of aging: 1 - slow aging, 2 - average aging, 3 - accelerated aging, 4 - long-lived women.

The results of canonical analysis are given in Figure 1. Four central points for the four studied groups of women with different tempos of aging are presented on this graph. The first canonical variable (X-axis) at its positive pole isolated a group of long-lived women with high values of heart rate, high waist-hip ratio, and high values of specific metabolic rate (see Table 5). Very low values of handgrip, low BMI, and relatively low amounts of fat and muscle mass are also typical for this group. Women with slow tempos of aging are located at the opposite end of the first canonical variable (the youngest according to their biological age).

An important result of this analysis refers to the location of the group with accelerated aging. This category is characterized with high BMI, excessive amount of fat mass, decrease in skeletal muscle mass, and low values of hand grip strength (see Table 5). A rather isolated location of this group indicates an unfavorable state of the organism and a potentially shorter lifespan.

The fact that women with accelerated tempos of aging approach those from long-lived group with regards to morphofunctional characteristics (see Tables 3, 4, 5, and Figure 1) serves as evidence that early-aging women with an average chronological age of 66 years, due to the level of involutive changes (progressive aging) are ahead of their counterparts of the same chronological age and closer to those of 90-year-olds. In other words, the degree of ‘wear and tear’ of their morphofunctional systems is very high and the probability to reach longevity for them is very low.

Canonical discriminant analysis showed a number of morphofunctional characteristics that are associated with early aging. It is particularly important that women with early aging are differentiated from women with normal (average) aging using these traits, although the chronological age in those two groups is the same (66 years). Thus, the combination of such traits can be a predictor of early aging.

## Conclusions

The results of this study show close connections between morphofunctional changes, particularly in body mass components and biological age.

The software ‘Diagnostics of Aging. BioAge’ (National Gerontological Center, Moscow, Russia, http://www.ngcrussia.org/) demonstrated its validity in the estimation of biological age in the group of elderly women (60 to 74-years-old). In the homogenous (according to their chronological age) group of women three subgroups were separated by different tempos of aging: lower rates of aging (biological age was less than chronological age by two years or more); consistent with their chronological age, and accelerated tempos of aging (biological age is higher than chronological years by two years or more).

Characteristics of body composition, cardiovascular system and power capacity demonstrate the trends of age involutive changes which can be traced from the group with slow rates of aging to the group with average rates, and then to the group with accelerated tempos of aging, and finally to the long-lived group. The results of comparative analysis show that women with accelerated aging are characterized with such traits as lower skeletal muscle mass, lower hand grip strength, and a higher metabolic rate.

Canonical discriminant analysis revealed a number of morphofunctional characteristics which differentiate early-aging women from women with average rates of aging. Among them are higher BMI values, excessive fat mass, lower skeletal muscle mass, and low values of hand grip strength. Thus the presence of such characteristics in elderly women can be considered as additional risk factor towards early onset of the aging process.

It can be suggested that those who demonstrate progressive signs of aging are in need of preventive and rehabilitation measures and further medical treatment.

## Consent

Written informed consent was obtained from the patients for the publication of this report and any accompanying images.

## Abbreviations

BA: Biological age; BMI: Body mass index; BM: Body mass; Ht: Height; FFM: Fat-free mass; FM: Fat mass; BCM: Body cell mass; SMM: Skeletal muscle mass; BMR: Basal metabolic rate; PBA: ‘Proper biological age’.

## Competing interests

The authors declare that they have no competing interests.

## Authors’ contributions

MN was head of the project, the investigation of elderly women (aging 60 - 74 years), statistical analysis, and writing up the results. NL was involved in the investigation of women from the longevity group (over 90), analysis of literature, data analysis, and writing up the results. RO was involved in the organization of the survey of women in Tiraspol, collection of questionnaire data, and discussion of the results. EG was involved in the discussion and writing up the results (English translation). All authors read and approved the final manuscript.
